# Supplementary material for: How much does it cost to measure immunity? A costing analysis of a measles and rubella serosurvey in southern Zambia
Source: PLoS One. 2020 Oct 15;15(10):e0240734. doi: 10.1371/journal.pone.0240734 (PMC7561102; doi:10.1371/journal.pone.0240734)
Supplement: S1 File — (DOCX) [file pone.0240734.s001.docx]

Serosurvey sample size calculations

Our base case of the nested serosurvey used to estimate costs had 16 clusters with an average of 43 participants enrolled per cluster (SD=17). The intraclass correlation was 0.026 for measles, with a mean seroprevalence of 96.5% (SD=5.1). For rubella, it was 0.005, with a mean seroprevalence of 95% (SD=6.0). The sample size calculations for this serosurvey assumed 85% estimated population immunity with +/-7% precision and a design effect of 1.4, resulting in 16 clusters with 12 children per cluster.

Cost ratio of cluster to participant cost

The cost ratio of marginal cluster cost to marginal participant cost was 54, reflective of a much higher cost to add a cluster of average size compared to adding an additional participant within an existing cluster. The cost ratio can guide when it may be more efficient to add an additional participant versus an additional cluster [23]. In this serosurvey, it was more cost efficient to add an additional participant in a cluster. However, this has to be weighed against the information provided by including an additional participant. If a cluster is fairly homogenous, adding a participant may not provide more information; conversely, if a cluster is heterogeneous, it could provide valuable information. This would be guided by the intraclass correlation.
