# Supplementary material for: How much does it cost to measure immunity? A costing analysis of a measles and rubella serosurvey in southern Zambia
Source: PLoS One. 2020 Oct 15;15(10):e0240734. doi: 10.1371/journal.pone.0240734 (PMC7561102; doi:10.1371/journal.pone.0240734)
Supplement: Attachment — Submitted filename: Response to reviewers.docx [file pone.0240734.s003.docx]

**Response to reviewers**

Thank you kindly for the thorough review of the manuscript. We have reviewed the comments and provided responses below in blue. Additionally, we have updated the manuscript, and noted where in the manuscript the changes are reflected. Line numbers are based on unmarked version.

1. Please ensure that your manuscript meets PLOS ONE's style requirements, including those for file naming. The PLOS ONE style templates can be found at https://journals.plos.org/plosone/s/file?id=wjVg/PLOSOne_formatting_sample_main_body.pdf and https://journals.plos.org/plosone/s/file?id=ba62/PLOSOne_formatting_sample_title_authors_affiliations.pdf

This has been updated.

2. We note that you have indicated that data from this study are available upon request. PLOS only allows data to be available upon request if there are legal or ethical restrictions on sharing data publicly. For information on unacceptable data access restrictions, please see http://journals.plos.org/plosone/s/data-availability#loc-unacceptable-data-access-restrictions.

In your revised cover letter, please address the following prompts:

a) If there are ethical or legal restrictions on sharing a de-identified data set, please explain them in detail (e.g., data contain potentially identifying or sensitive patient information) and who has imposed them (e.g., an ethics committee). Please also provide contact information for a data access committee, ethics committee, or other institutional body to which data requests may be sent.

b) If there are no restrictions, please upload the minimal anonymized data set necessary to replicate your study findings as either Supporting Information files or to a stable, public repository and provide us with the relevant URLs, DOIs, or accession numbers. Please see http://www.bmj.com/content/340/bmj.c181.long for guidelines on how to de-identify and prepare clinical data for publication. For a list of acceptable repositories, please see http://journals.plos.org/plosone/s/data-availability#loc-recommended-repositories.

We will update your Data Availability statement on your behalf to reflect the information you provide.

The minimal anonymized data set necessary to replicate our study findings has been uploaded as supporting files.

3. Thank you for stating the following in the Competing Interests section:

'AC, KH, WM, and BP received financial support from the Bill & Melinda Gates Foundation. Other authors declare that they have no competing interests'

Please confirm that this does not alter your adherence to all PLOS ONE policies on sharing data and materials, by including the following statement: "This does not alter our adherence to  PLOS ONE policies on sharing data and materials.” (as detailed online in our guide for authors [http://journals.plos.org/plosone/s/competing-interests](about:blank)).  If there are restrictions on sharing of data and/or materials, please state these. Please note that we cannot proceed with consideration of your article until this information has been declared.

Please include your updated Competing Interests statement in your cover letter; we will change the online submission form on your behalf.

Please know it is PLOS ONE policy for corresponding authors to declare, on behalf of all authors, all potential competing interests for the purposes of transparency. PLOS defines a competing interest as anything that interferes with, or could reasonably be perceived as interfering with, the full and objective presentation, peer review, editorial decision-making, or publication of research or non-research articles submitted to one of the journals. Competing interests can be financial or non-financial, professional, or personal. Competing interests can arise in relationship to an organization or another person. Please follow this link to our website for more details on competing interests: [http://journals.plos.org/plosone/s/competing-interests](about:blank)

This is correct. It did not alter our adherence to PLOS ONE policies on sharing data and materials. We added this to the cover letter.

Reviewer #1: Thank you for giving me the opportunity to review this interesting paper. I think the topic is relevant and based on sound data. However, I have several concerns regarding the presentation.

Abstract:
Background: the reason why serosurveys are valuable is not clearly stated.

This has been added (line 21).
Methods are more detailed than required

Some of the details have been removed (lines 27-28).
Results: Please see my comment to Results, line 214 to 223.

This has been clarified (lines 34-39).

Introduction: The introduction is hard to read as it seems to be a collection of single one-sentence statements that lack context. Thus, the reason why the study was performed remains somewhat unclear. The aim of the study should be described better. To me, the phrase “cost integrated vaccine-preventable disease surveillance that includes calculating serosurvey” is not understandable.
The introduction has been reworked to be more cohesive. The aim of the study is more clearly described in the last paragraph, with the clarification on the phrase noted (lines 85-90).

Methods:
Line 105 to 106: “Clusters were defined as administrative boundaries known as Supervisory Enumeration Area” – what does that mean?

An Enumeration Area (EA) is a small geographic unit below a ward in urban areas and village in rural areas, into which the whole country is divided for census purposes. A supervisor is responsible for accounting for all of the people within their assigned enumeration areas. We tried to clarify that it is a small geographic area. (lines 100-102)

Line 112-115: Meaning unclear.
This has been modified. The intent was to explain that a bottom-up approach was used to cost each item required for the serosurvey. We included costs that were incurred by the government healthcare system as they implemented the serosurvey but did not include participant costs. (lines 107-110)

Line 133 to 137: Meaning unclear.
The text has been updated to be clearer. The intent was to explain Figure 1 and how the phases and categories were developed. (lines 127-132)

Line 155-57: Surprisingly high amount of Man-power to collect specimens, 3 working days à five persons to collect 41 specimens. Please explain why teams contain five persons.
The teams consisted of 5 people-1 supervisor, 1 phlebotomist, and 3 data collectors (one who was a back-up phlebotomist). This is the actual number of people that went out to the field for the serosurvey. Because this serosurvey was nested within a vaccination coverage survey, the teams conducting the serosurvey were asked to work at the same pace as those doing only the vaccination coverage survey. Whereas teams doing only the vaccination coverage survey in other clusters only needed to interview mothers of 12 children; serosurvey teams had to both interview and collect blood from everyone in the household of those 12 children. The mean number of participants enrolled in each household was 4 (IQR: 3-6) [1]. This meant that additional staff were required to complete all of the work within the allotted 2 weeks in the field. If more time had been allowed, the teams could have been smaller.

Line 174.175: Why were community health care workers invited to accompany the working team, thus enhancing the costs?

Community health workers accompanied the teams for several reasons. They are familiar with the area and could guide the teams in interpreting the maps. And they could make appropriate introductions to community leaders so the community was more likely to participate in the study. [2] (Lines 167-171)

Results:
Lien 214 to 223: I don’t understand the presentation of the percentages. In total 220 % are presented. How are they broken down?
The paragraph has been reorganized to better clarify. The percentages are presented in a couple of formats: by phase of serosurvey implementation (as broken down in Table 1) and by input category (as denoted in Figure 2). This corresponds with the rows and columns from Figure 1. Additionally, lines 214-217 explain the costs per category input for just adding the serosurvey to the already planned coverage survey (the different colors in Figure 2). (Lines 209-218)

Line 237 to 239: I do not see the relevance of this information.
Cost ratio information has been moved to supplemental material.

Line 240 to 242: Incomplete sentence.
This sentence has been split into two and moved to supplemental material.

Line 242-243: Discussion of results
Cost ratio information has been moved to supplemental material.

Line 244 to 250: Very detailed information, but unclear relevance.
This has been condensed to get across the main points more clearly. The relevance was intended to denote that by adding additional children in the serosurvey than were in the vaccination coverage survey, there was an additional cost. Similarly, we included adults in the serosurvey sample, which also had an additional cost. The implications of this are in the discussion section (lines 295-304), highlighting that an immunity gap in women of childbearing age was identified through this serosurvey because adults were included.

Sensitivity analysis: I do not understand how the sensitivity analysis was performed.
One-way sensitivity analyses were done to see how changing parameters for the serosurvey would have affected the cost. For example, decreasing the number of team members to 2 resulted in a $5,000 cost savings, whereas increasing the number of team members to 6 resulted in increased costs. This was similarly done for time spent in the field, training, planning, and laboratory testing. The footnote for Figure 3 indicates what the ranges for the sensitivity analyses were. (lines 252-258)

Discussion:
No overview of the main results
An initial paragraph has been added to provide an overview and the value of serosurveys in guiding immunization programs (lines 261-269).

Relevance of the study: It is not discussed how measuring seronegativity contributes to public health, or how the costs for a serosurvey relate to cost of other measurements to enhance public health, like vaccination campaigns or nutrition supplements.
We agree this is a really important question, but unfortunately we were not able to address it in this study. We are working through our research to better understand the added value of targeted serosurveys and how they could be used to guide immunization programs. In future research we would do a cost-benefit or cost-utility analysis to be able to compare the costs of a serosurvey to those of alternative strategies. Alternative strategies could include approximate measurements of seropositivity using vaccination coverage and fever rash surveillance systems,. This could also compare a targeted serosurvey with mop-up immunization in areas found to have low seroprevalence to a blanket vaccination campaign (lines 325-329).

Line 326 to 327: Incomplete& grammatically wrong sentence.
Thank you for catching this. It has been updated.

Line 330: not included into the pdf
We are not sure what this is in reference to, but the minimal dataset has now been made available.

Line 334 to 337: Unclear why this is a weakness
It was not intended to be a weakness, but rather a limitation in terms of generalizability. In our study, we hired new personnel. If this were integrated as an ongoing activity that forms part of the existing surveillance system, it could be done by existing staff. This could result in lowered costs and would make it more sustainable in the long run, rather than hiring and training new personnel. (Lines 396-399)

Acknowledgement: One of the co-authors is acknowledged for his contribution.
This has been removed and updated (line 352).

Figures: The figures are of insufficient quality in the pdf-file provided.

These have been updated.

Reviewer #2: Thanks for the manuscript and it read very well. Please see the following comments for further improvement of the manuscript.
1. Line 244 to 248 should be in methods section for better clarity including the age range for serology survey.

This line is intended to be a reminder for the reader as to why we are saying “additional” children and what the cost was for adding adults. We have tried to make this clearer in the methods section. (lines 97-98)

2. Line 251 to 256 would be clear if the author presents as a table in result section.
A table has been added to the results section (line 244-246).

3. The authors stated in the method section that 12 children were selected from each cluster. But in line 293 to 295 stated the fact about women of child bearing age which do not seem to be in this survey. Please clarify this fact.
The post-coverage evaluation survey selected one child from 12 different households in each cluster. However, our nested serosurvey collected specimens from all household members regardless of age, not just the child. Therefore, we were able to calculate seroprevalence estimates for women of childbearing age.

4. It would be more informative if author could provide the administration of vaccination coverage in the survey area to argue with needs to conduct serology survey.
Administrative vaccination coverage for the 2016 measles-rubella vaccination campaign was 89.9% (95% confidence interval (CI): 85.9, 92.8) for 9 month to 16 year olds. By comparison, the serosurvey prevalence was 96.1% (95% CI: 92.4, 98.1) for measles and 98.4% (95% CI: 95.9, 99.4) for rubella for the same age range. For measles, the higher seroprevalence was likely due to the fact that many children had already been vaccinated through the routine immunization program. For rubella, this is likely because they had already been previously exposed to circulating rubella virus, as this was the first introduction of rubella vaccine. This justification for conducting serological surveillance has been added to the discussion (lines 286-294)

5. Figures in the manuscript is not clear and suggest to provide higher resolution figure.

These have been updated.

1.	Hayford, K., et al., *Measles and rubella serosurvey identifies rubella immunity gap in young adults of childbearing age in Zambia: The added value of nesting a serological survey within a post-campaign coverage evaluation survey.* Vaccine, 2019. **37**(17): p. 2387-2393.

2.	Mutembo, S., et al., *Integrating Blood Collection within Household Surveys: Lessons Learned from Nesting a Measles and Rubella Serological Survey within a Post-Campaign Coverage Evaluation Survey in Southern Province, Zambia.* Am J Trop Med Hyg, 2018. **99**(6): p. 1639-1642.
